# RACGAP1 promotes lung cancer cell proliferation through the PI3K/AKT signaling pathway

**DOI:** 10.1038/s41598-024-58539-0

**Published:** 2024-04-15

**Authors:** Zhiyang Xu, Shaohang Wu, Jiahua Tu, Mingyang Wang, Weicheng Liang, Jiangdong Cheng, Jun Guan, Jianxin Xu

**Affiliations:** Department of Thoracic Surgery, The First Hospital of Putian, The School of Clinical Medicine, Fujian Medical University Putian, Fujian, 351100 China

**Keywords:** Lung adenocarcinoma, RACGAP1, Survival, Proliferation, Apoptosis, Cancer genetics, Gastrointestinal cancer

## Abstract

We aimed to investigate the expression and clinic significance of Rac GTPase Activating Protein 1 (RACGAP1) in human lung adenocarcinoma (LUAD). Online database analysis revealed a significant increase in RACGAP1 mRNA expression among 26 types of tumor tissues, including LUAD tissues. Online database and tissue microarray analyses indicated that RACGAP1 expression was significantly upregulated in LUAD tissues. Genetic variation analysis identified four different genetic variations of RACGAPs in LUAD. Moreover, online database analysis showed that RACGAP1 upregulation was correlated with shorter survival in patients with LUAD. After silencing RACGAP1 expression in A549 cells using siRNA and assessing its protein levels via Western blotting, we found that RACGAP1 knockdown inhibited cell growth and induced apoptosis determined using the Cell Counting Kit-8 assay, colony formation assay, and flow cytometry. Mechanistically, western blot analysis indicated that Bax expression increased, whereas Bcl-2 expression decreased. Moreover, RACGAP1 knockdown attenuated PI3K/AKT pathway activation in lung cancer cells. Taken together, our findings showed that RACGAP1 was overexpressed in LUAD tissues and played an important role in lung cancer by increasing cell growth through the PI3K/AKT signaling pathway. This study suggests recommends evaluating RACGAP1 in clinical settings as a novel biomarker and potential therapeutic target for lung cancer.

## Introduction

Lung cancer, including non-small cell adenocarcinoma, squamous cell lung carcinoma, and large cell carcinoma, is the most common type of cancer among men and the third most common type of cancer among women^1,2^, with lung adenocarcinoma (LUAD) being the most common type of lung cancer. Based on 2020 global cancer statistics, lung cancer still remains the leading cause of cancer death in not only low- and middle-income countries but also most high-income countries^3^. The treatment of lung cancer depends on the cancer type and stage; however, most patients with lung cancer are diagnosed with advanced-stage disease, and molecular targeted therapies are only limited to specific patients^4^. Therefore, further exploration of molecular targets are essential for uncovering more innovative diagnostic, preventive, and treatment strategies.

Rac GTPase-activating protein 1 (RACGAP1), also called MgcRACGAP, had been first detected in 1998 from human testis and germ cell tumor extracts and plays an important role in the Rho GTPase activation cycle by regulating Rho GTPase transformation and GTP hydrolysis stimulation^5^. Studies have shown that RACGAP1 is involved in cytokinesis, induces cell proliferation, and might be related to the well-known proliferative marker Ki67^6–9^. Moreover, RACGAP1 has been found to play a role in cell transformation, migration, and metastasis^10–13^. These reported functions have attracted researcher attention to its potential role in malignancies^14^. Consequently, an increasing number of studies have reported an the association between RACGAP1 overexpression in several types of cancers, including stomach^15^, breast^16,17^, colorectal^18^, bladder^19^, liver^20^, and uterine cancers^21^, indicating its possible role in tumor proliferation, migration, and recurrence. However, no detailed evidence has been available on the relationship between RACGAP1 and lung cancer diagnosis and prognosis, except for its upregulation in lung cancer^22,23^.

The present study used bioinformatics analysis of online databases and tissue microarray (TMA) to evaluate the expression profile of RACGAP1 and the relationship between RACGAP1 expression and clinical pathological parameters in lung cancer. Moreover, a series of experiments using representative lung cancer cell line were conducted to examine the functional role of RACGAP1.

## Results

### Upregulation of RACGAP1 in LUAD

**Figure 1:**
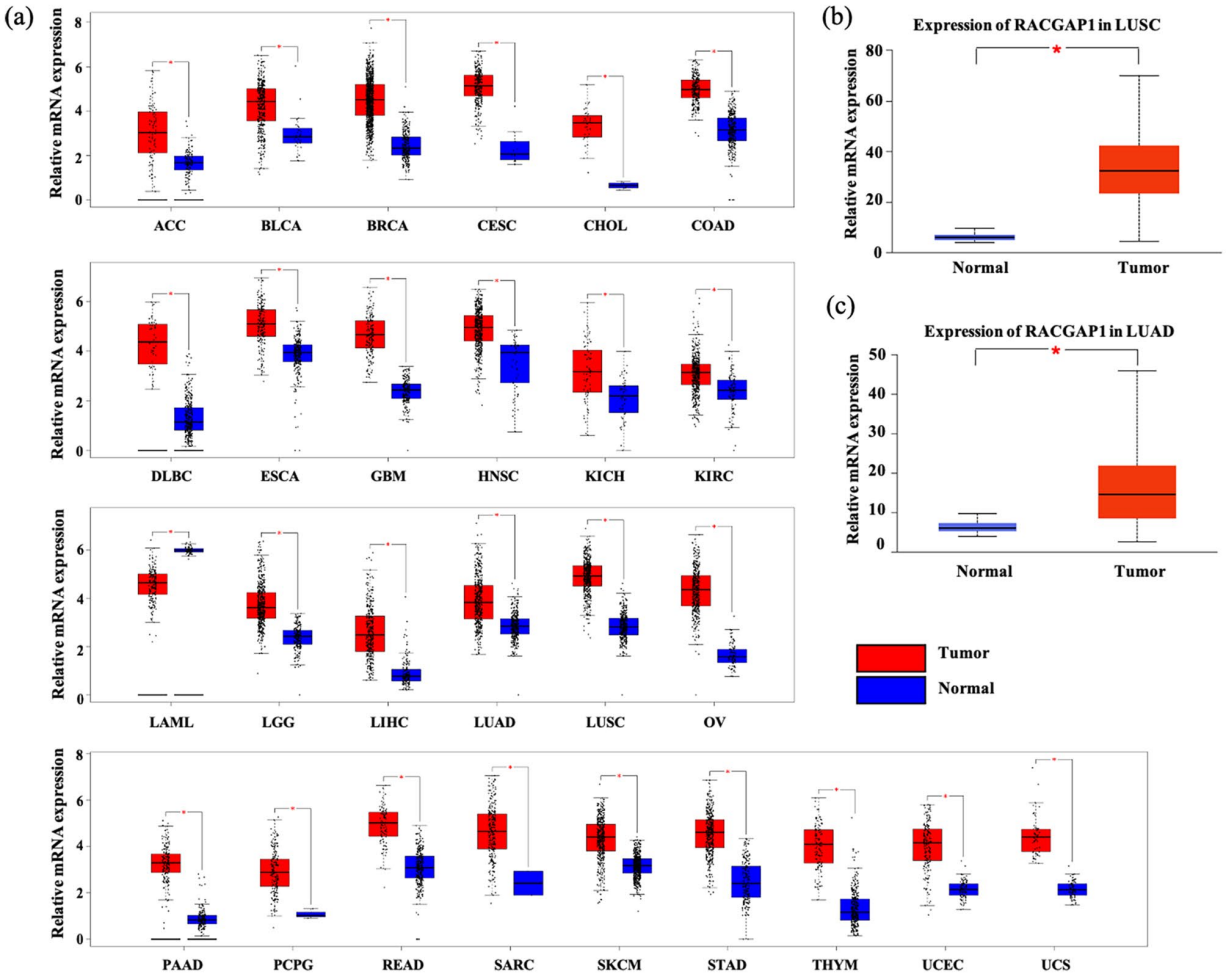
RACGAP1 mRNA expression levels in different human cancers. (**a**) The mRNA expression of RACGAP1 in different datasets of different cancers was analyzed using the GEPIA website. (**b**, **c**) RACGAP1 expression in LUAD and LUSC samples and paired normal samples analyzed through the UALCAN website. *P < 0.05 vs. normal tissues. *ACC* Adrenocortical Carcinoma, *BLCA* Bladder Carcinoma, *BRCA* Breast Invasive Carcinoma, *CESC* Cervical Carcinoma, *CHOL* Cholangiocarcinoma, *COAD* Colon Adenocarcinoma, *DLBC* Lymphoid Neoplasm Diffuse Large B-cell Lymphoma, *ESCA* Esophageal Carcinoma, *GBM* Glioblastoma Multiforme, *HNSC* Head and Neck Squamous Cell Carcinoma, *KICH* Kidney Chromophobe, *KIRC* Kidney Renal Clear Cell Carcinoma, *LAML* Acute Myeloid Leukemia, *LGG* Brain Lower Grade Glioma, *LIHC* Liver Hepatocellular Carcinoma, *LUAD* Lung Adenocarcinoma, *LUSC* Lung Squamous Cell Carcinoma, *OV* Ovarian Serous Cystadenocarcinoma, *PAAD* Pancreatic Adenocarcinoma, *PCPG* Pheochromocytoma and Paraganglioma, *READ* Rectum Adenocarcinoma, *SARC* Sarcoma, *SKCM* Skin Cutaneous Melanoma, *STAD* Stomach Adenocarcinoma, *THYM* Thymoma, *UCEC* Uterine Corpus Endometrial Carcinoma, *UCS* Uterine Carcinosarcoma.

**Figure 2:**
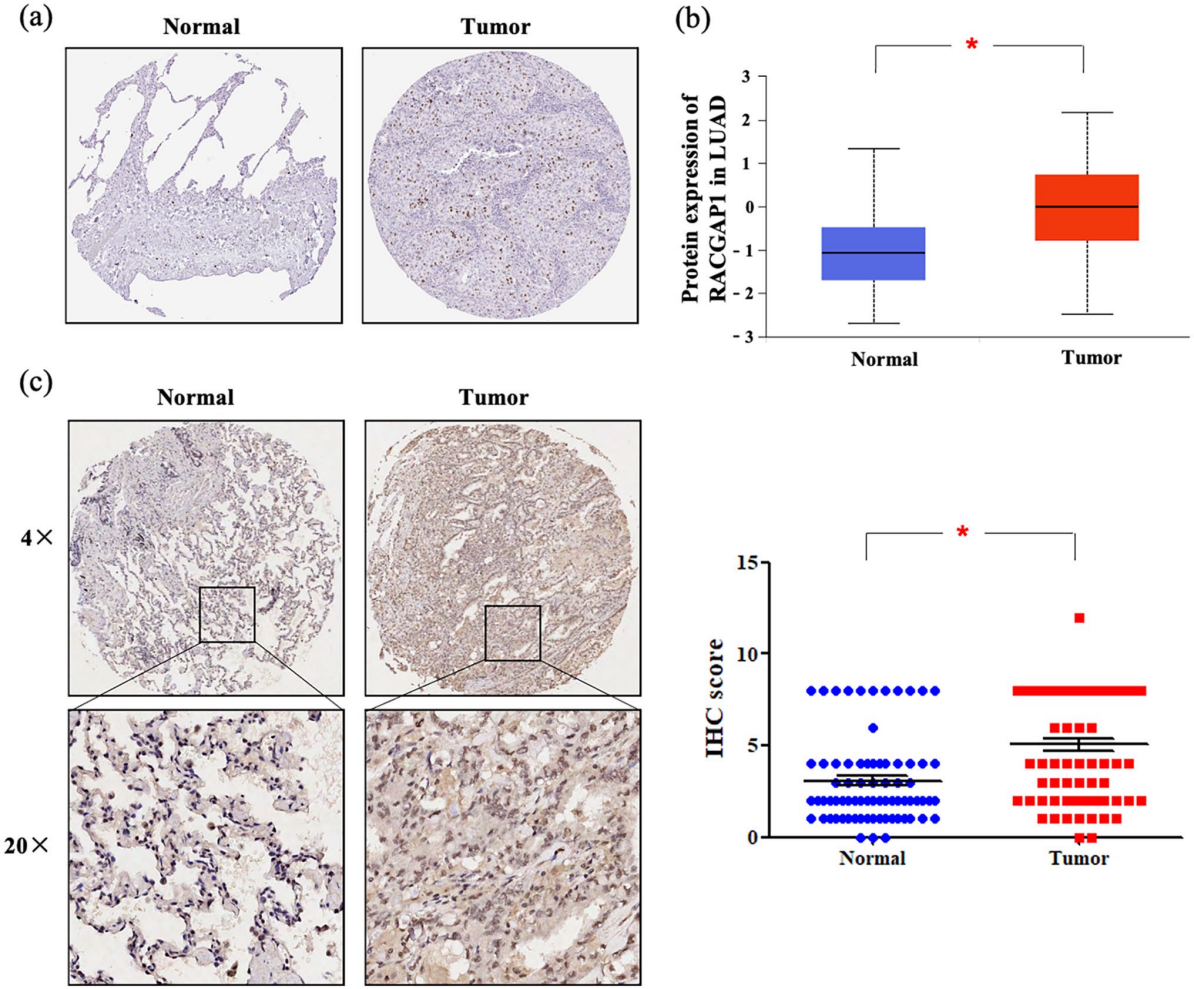
RACGAP1 protein expression in lung cancer tissues and normal tissues. (**a**) Immunohistochemical staining of RACGAP1 in LUAD tissues and normal lung tissues from the HPA database. (**b**) RACGAP1 protein expression in LUAD tissues and normal lung tissues from CPTAC samples. (**c**) RACGAP1 protein levels were determined via IHC using tissue microarrays in 75 pairs of lung adenocarcinoma and adjacent noncancerous lung tissues. The lower right panels show representative images captured at × 4 and × 20 magnification. Each datapoint in the plot represents one tissue sample. Error bars represent the median ± range. T, lung cancer tissue; N, noncancerous lung tissue. *P < 0.05 vs. normal tissues.

We compared RACGAP1 levels between various types of tumors and adjacent normal tissues using Gene Expression Profiling Interactive Analysis (GEPIA) databases. Our findings showed that although RACGAP1 mRNA expression was downregulated in acute myeloid leukemia, it was dramatically upregulated in most of tumors, including LUAD and lung squamous cell carcinoma (LUSC), compared to adjacent noncancerous tissues (Fig. 1a; *P* < 0.05). Consistently, data from the University of ALabama at Birmingham CANcer data analysis Portal (UALCAN) website sourced from The Cancer Genome Atlas (TCGA) confirmed that RACGAP1 mRNA expression was upregulated in both LUSC (Fig. 1b) and LUAD (Fig. 1c) tissues compared to that in the normal tissues (*P* < 0.05). We further investigated the protein levels of RACGAP1 in LUAD using the Human Protein Atlas (HPA) and UALCAN. As shown in Fig. 2a, we found weak or no RACGAP1 expression in normal lung tissues but moderate and high RACGAP1 expression in lung cancer tissues. Consistently, analysis of CPTAC samples using UALCAN also showed that RACGAP1 protein levels were significantly higher in in LUAD tissues than in normal lung tissues (Fig. 2b, *P* < 0.05). We then examined RACGAP1 expression in surgically resected tumors from 75 patients with lung cancer using immunohistochemistry (IHC). The clinicopathological features of lung adenocarcinoma patients are summarized in Table 1. RACGAP1 expression was significantly greater in tumor tissues than in adjacent normal tissues. A significant difference in RACGAP1 staining pattern was observed between the two groups (Fig. 2c, *P* < 0.05). The aforementioned findings revealed that RACGAP1 was upregulated in LUAD at both the mRNA and protein levels.

**Table 1:** Clinicopathological features of 75 lung adenocarcinoma patients in tissue microarray.

Characteristics	n (%)
Age (years)	
< 65	52 (69)
≥ 65	23 (31)
Sex	
Females	33 (44)
Males	42 (56)
Tumor size	
≤ 5 cm	49 (65)
> 5 cm	26 (35)
Clinical stage	
I	0 (0)
II	48 (64)
III	27 (36)
IV	0 (0)
T stage	
T1	15 (20)
T2	34 (45)
T3	25 (33)
T4	1 (2)
N stage	
N0	41 (55)
N1	12 (16)
N2	22 (29)
M stage	
M0	73 (97)
M1	2 (3)
Lymph node metastasis	28 (37)
Distant metastasis	2 (3)

### Genetic alterations of RACGAP1 in LUAD

**Figure 3:**
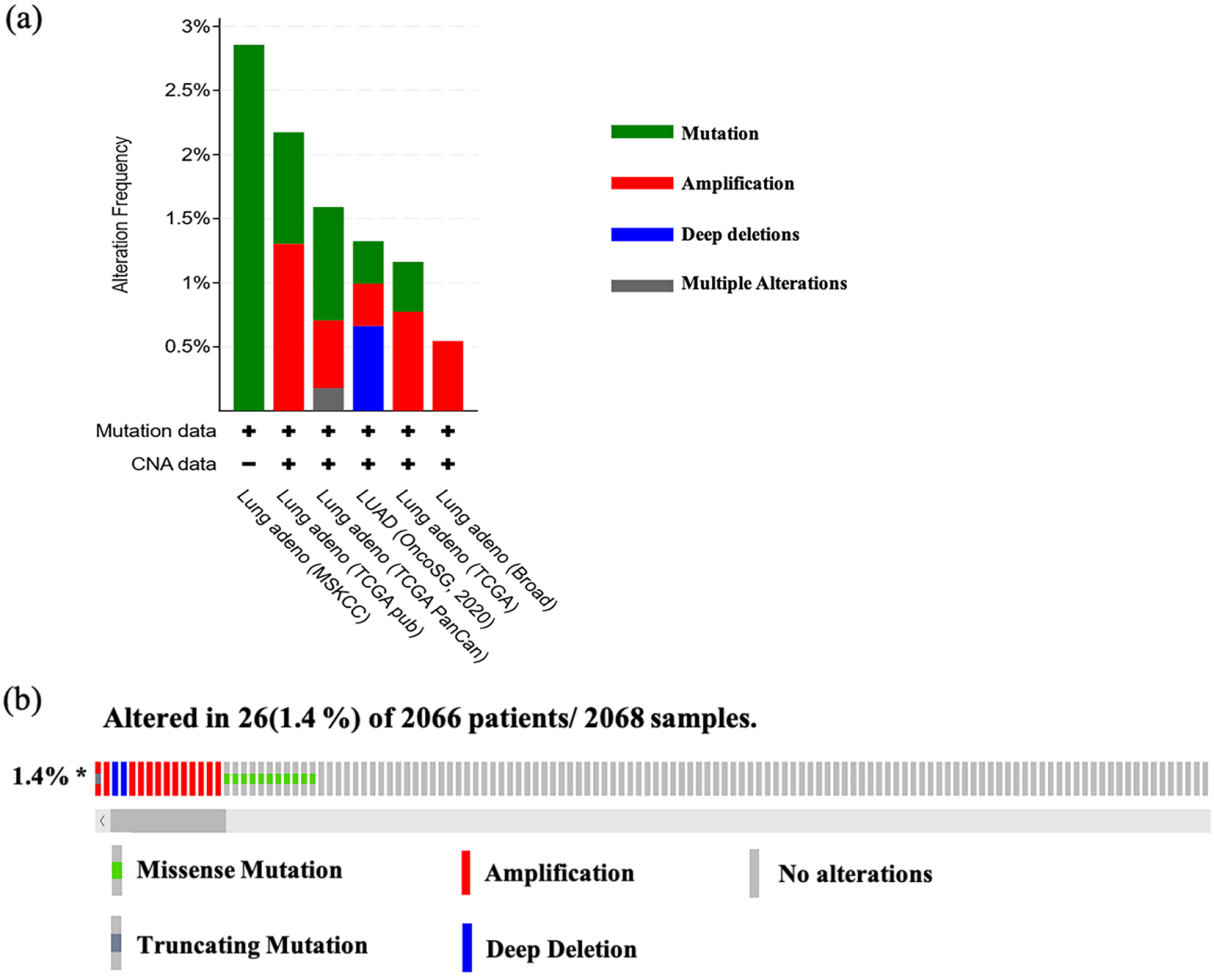
Analysis of RACGAP1 alterations in LUAD. (**a**) CNA and mutation frequency data for RACGAP1 in LUAD were analyzed through the cBioPortal website. (**b**) OncoPrint visual summary of alterations on a query of RACGAP1. Variations in frequency included missense mutation (green), truncating mutation (gray), amplification (red), and deep deletions (blue).

Genetic variations of RACGAP1 in 2068 cases retrieved from 7 studies (35 cases from MSKCC; 230 cases from TCGA pub; 566 cases from TCGA, PanCancer; 302 cases from OncoSG, 2020; 516 cases from TCGA; 183 cases from Broad; and 0 cases from TSP) were analyzed using the cBioPortal database (Fig. 3a). We found four different genetic variations of RACGAP1 in LUAD, including missense mutation, truncating mutation, amplification, and deep deletion (Fig. 3b). Most genetic variations of RACGAPS were missense mutations and amplifications (incidence rates of 0.53% and 0.58%, respectively).

### Relationship between RACGAP1 expression and clinicopathological parameters of LUAD patients

**Figure 4:**
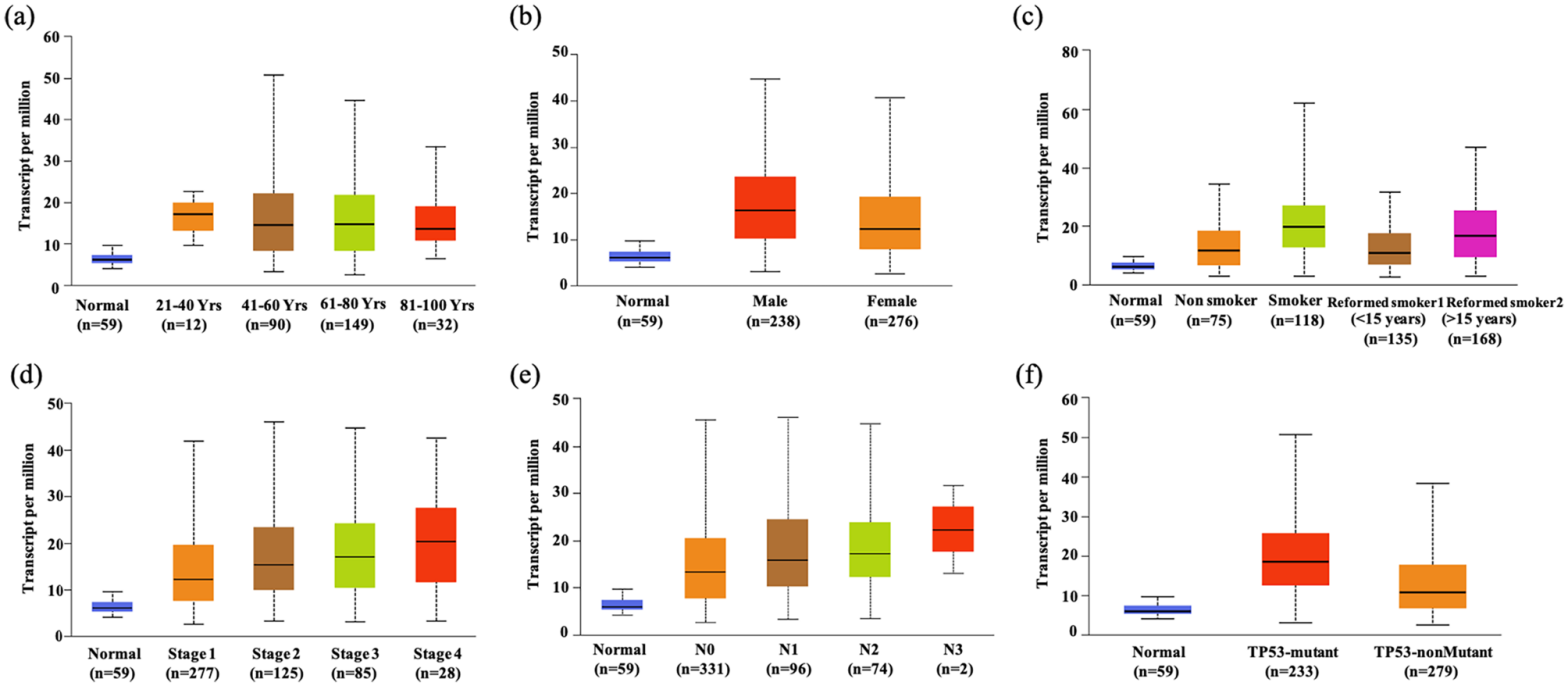
Correlation between RACGAP1 expression and clinical features of LUAD patients. Correlation between RACGAP1 expression and (**a**) age, (**b**) sex, (**c**) smoking habits, (**d**) stage, (**e**) nodal metastasis, or (**f**) TP53 mutation status of LUAD patients.

To investigate the correlation between RACGAP1 expression and clinicopathological parameters of patients, the UALCAN database was used to obtain information regarding age, sex, smoking habits, disease stage, nodal metastasis, and p53 mutants, after which their relationship with RACGAP1 levels were determined. Interestingly, all LUAD patients exhibited increased RACGAP1 expression levels (Fig. 4a; 41–60, 61–80, or 81–100 vs. normal, all *P* < 0.05). Moreover, we found that both male and female patients had increased RACGAP1 mRNA expression compared to normal and that male patients had increased RACGAP1 mRNA expression compared to female patients (Fig. 4b; *P* < 0.05). Furthermore, we observed higher RACGAP1 mRNA levels in smokers than in nonsmokers, reformed smokers (< 15 years), and reformed smokers (> 15 years) (Fig. 4c; all *P* < 0.05). Moreover, a comparison between smokers and reformed smokers showed lower levels among the latter (smokers vs. reformed smokers > 15 years or < 15 years; both *P* < 0.05). In addition, RACGAP1 expression levels were increased across different stages of LUAD (Fig. 4d; compared to normal lung tissues, all *P* < 0.05), with late-stage tumor tissues exhibiting higher expression levels than did early-stage tumor tissues (Fig. 4d; stage 4 vs. stage 1; *P* < 0.05). Notably, we also found that RACGAP1 expression was higher in N0-, N1-, or N2-stage LUAD than in normal tissues (Fig. 4e, all *P* < 0.05). Additionally, TP53 mutants exhibited higher RACGAP1 levels than did normal tissues or TP53 non-mutants (Fig. 4f; all *P* < 0.05). Differences in the clinicopathological parameters are summarized in Table 2. Our results revealed that high RACGAP1 expression was significantly associated with sex, smoking habit, disease stage, nodal metastasis, and TP53 mutant status.

**Table 2:** Clinicopathological features of patients.

Clinicopathological features	P value
Age	
Normal-vs.-age (41–60 years)	1.62E−12***
Normal-vs.-age (61–80 years)	1.62E−12***
Normal-vs.-age (81–100 years)	1.15E−04***
Gender	
Normal-vs.-male	< 1E−12***
Normal-vs.-female	< 1E−12***
Male-vs.-female	1.93E−02*
Smoking habit	
Normal-vs.-nonsmokers	3.28E−10***
Normal-vs.-smokers	< 1E−12***
Normal-vs.-reformed smokers1	1.63E−12***
Normal-vs.-reformed smokers2	< 1E−12***
Nonsmokers-vs.-smokers	2.67E−07***
Nonsmokers-vs.-reformed smokers2	1.56E−04***
Smokers-vs.-reformed smokers1	1.90E−07***
Smokers-vs.-reformed smokers2	1.29E−02*
Reformed smoker1-vs-reformed smoker2	7.95E−05***
Individual cancer stages	
Normal-vs.-stage1	< 1E−12***
Normal-vs.-stage2	< 1E−12***
Normal-vs.-stage3	7.01E−14***
Normal-vs.-stage4	1.75E−05**
Stage1-vs.-stage4	1.42E−02*
Nodal metastasis status	
Normal-vs.-N0	< 1E−12***
Normal-vs.-N1	1.63E−12***
Normal-vs.-N2	6.28E−13***
TP53 mutant status	
Normal-vs.-TP53-mutant	< 1E−12***
Normal-vs.-TP53-nonmutant	1.62E−12***
TP53-mutant-vs.-TP53-nonmutant	1.27E−08***

*P < 0.05, **P < 0.01, ***P < 0.001.

### Upregulation of RACGAP1 was correlated with shorter survival in LUAD patients

**Figure 5:**
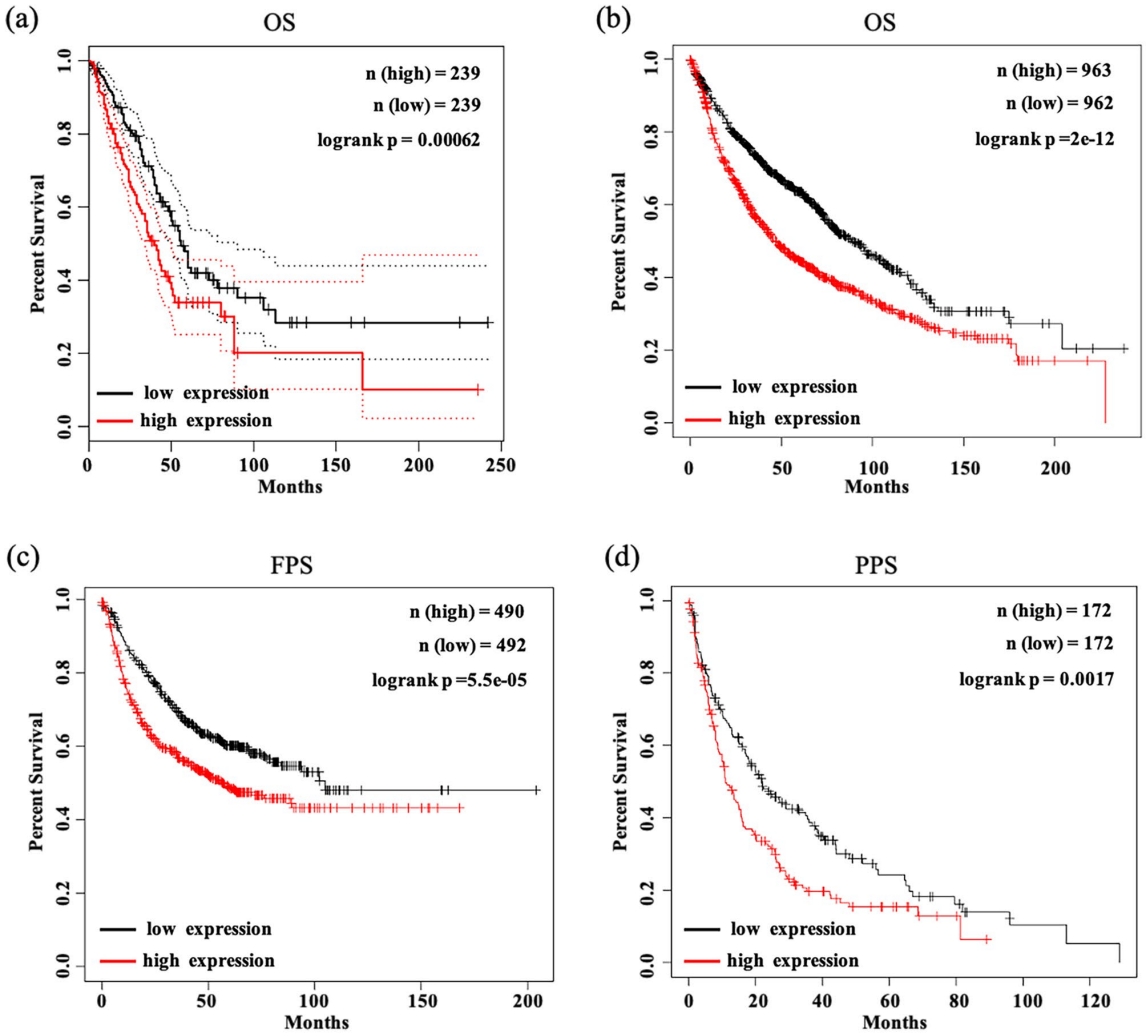
Correlation between RACGAP1 expression and survival of LUAD patients. (**a**) The correlation between RACGAP1 expression and OS of LUAD patients was analyzed using GEPIA. (**b**–**d**) The correlation between RACGAP1 expression and OS, PFS, or PPS of LUAD patients was analyzed through the Kaplan–Meier Plotter. OS, overall survival; PFS, free-progression survival; PPS, post-progression survival.

The current study further determined whether RACGAP1 was correlated with LUAD patient survival through both GEPIA and Kaplan–Meier survival curves. As shown in Fig. 5a, GEPIA indicated that increased RACGAP1 expression was correlated with decreased overall survival in LUAD patients (*P* = 1.70E-04). Consistently, analyses using the Kaplan–Meier Plotter revealed that increased RACGAP1 expression was significantly correlated with decreased overall survival (OS; Fig. 5b; hazard ratio [HR] 1.58, log-rank *P* = 2.0E−12), progression-free survival (PFS; Fig. 5c; HR 1.48, log-rank *P* = 5.5E−05), and post-progression survival (PPS; Fig. 5d; HR 1.5, log-rank *P* = 1.7E−03). These findings suggest the potential role of RACGAP1 as a biomarker for predicting prognosis.

### RACGAP1 knockdown inhibits A549 cell growth

**Figure 6:**
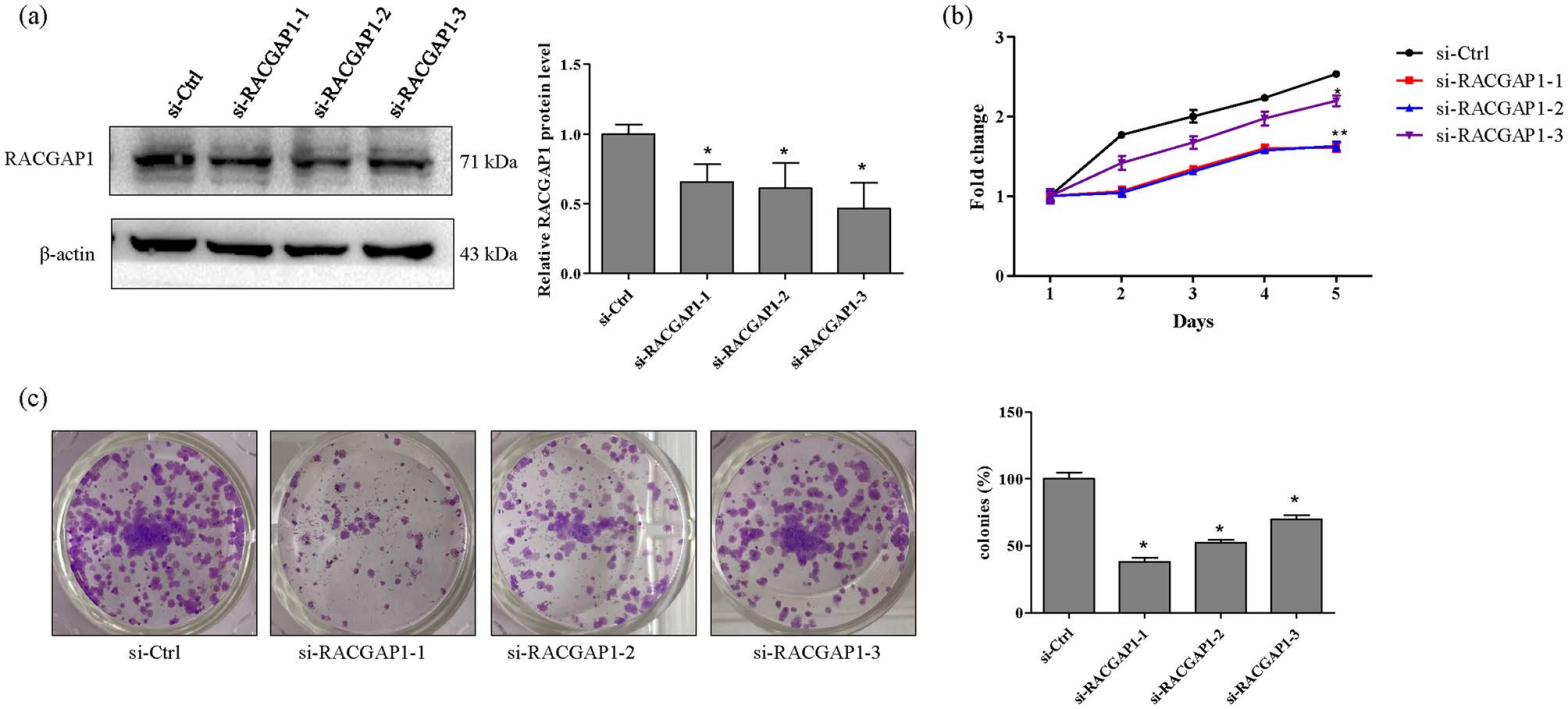
RACGAP1 knockdown inhibited cell growth in A549 cells. A549 cells were transfected with si-Ctrl or three independent siRNAs for si-RACGAP1. (**a**) Protein levels of RACGAP1 were determined using Western blot analysis. Representative images of RACGAP1 and β-actin are shown and quantitated using ImageLab software. β-actin was used as an internal control. *P < 0.05 vs. si-Ctrl. (**b**) Cell viability was determined using the CCK-8 assay. Data were normalized to the viability on day 1 and are represented as fold change. *P < 0.05 vs. si-Ctrl. (**c**) The colony formation assay was used to determine cell survival. After obtaining the images, colony formation was calculated and normalized to the survival of control cells. *P < 0.05 vs. si-Ctrl.

**Figure 7:**
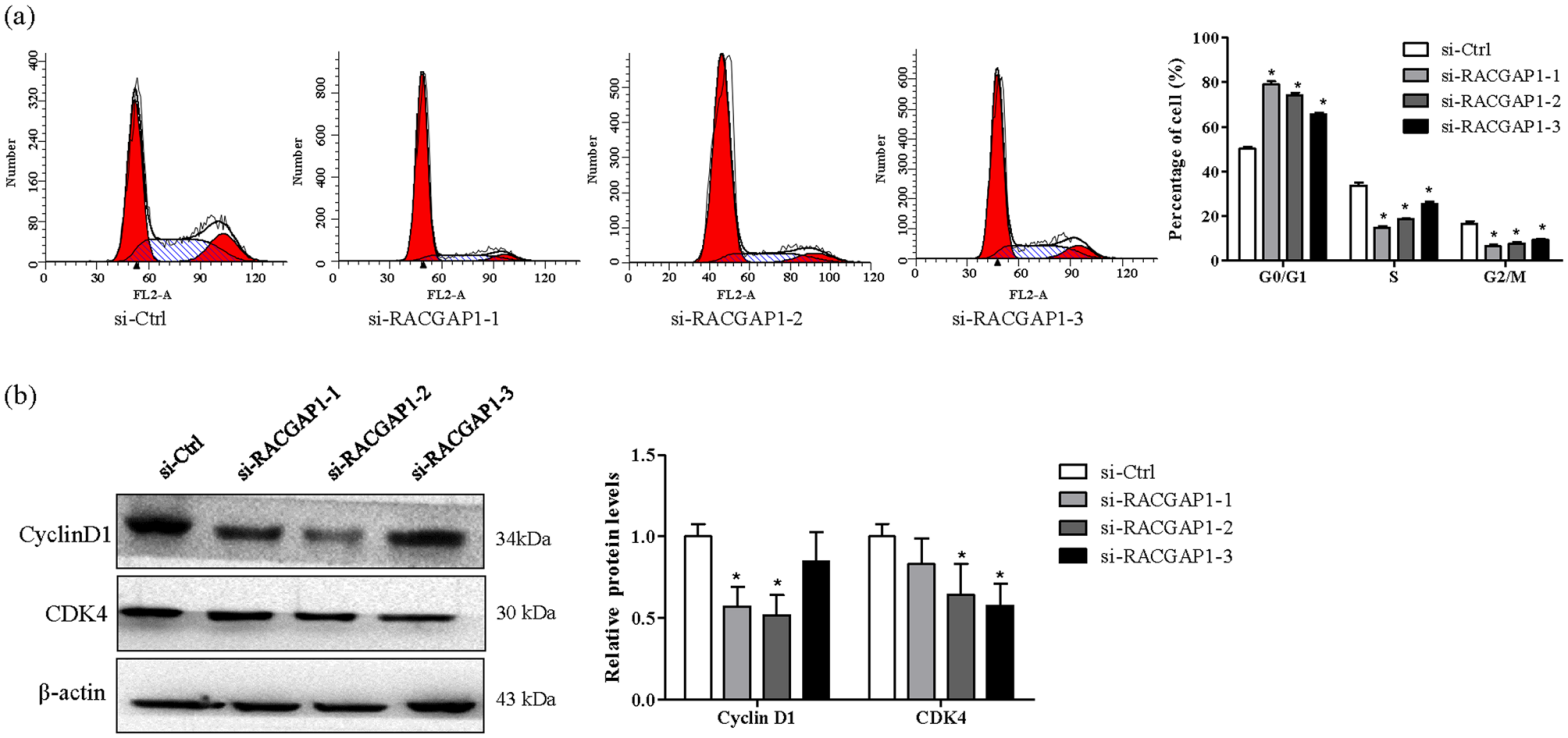
RACGAP1 knockdown inhibited cell proliferation in A549 cells. A549 cells were transfected with si-Ctrl or three independent siRNAs for si-RACGAP1. (**a**) Cell cycle progression was determined through PI staining and FACS analysis. Representative plots and percentage of cells at different stages (G0/G1, G2/M, and S phases) were presented. *P < 0.05 vs. si-Ctrl. (**b**) Protein levels of CDK4 and CyclinD1 after RACGAP1 knockdown were determined using Western blot analysis. Representative images are shown and quantitated using ImageLab software. β-actin was used as an internal control. *P < 0.05 vs. si-Ctrl.

To explore the biological function of RACGAP1 on LUAD cell growth, three independent siRNAs targeting RACGAP1 were designed, and knockdown efficiencies were determined using Western blotting. Notably, Western blot analyses revealed that RACGAP1 expression was significantly downregulated at the protein level in A549 cells with all three siRNAs (Fig. 6a). Next, we used transfected with A549 cells to evaluate the effects of RACGAP1 on cellular functions. As shown in Fig. 6b, RACGAP1 knockdown reduced the viability of A549 cells. Moreover, RACGAP1 knockdown inhibited the formation of colonies (Fig. 6c). To further elucidate the possible mechanism underlying cellular growth inhibition induced by RACGAP1 knockdown, the cell cycle assay was performed using flow cytometry. As shown in Fig. 7, RACGAP1 knockdown markedly increased the percentage of cells in the G0/G1 phase but decreased the percentage of cells in the S phase (Fig. 7a), as well as significantly downregulated the expression of G0/G1-related proteins CyclinD1 and CDK4 in A549 cells, excepted for si-RACGAP1-3 in Cyclin D1 and si-RACGAP1 inCDK4 (Fig. 7b). These results suggest that RACGAP1 knockdown may inhibit proliferation by inducing G0/G1 phase arrest.

### RACGAP1 knockdown induces apoptosis of A549 cells

**Figure 8:**
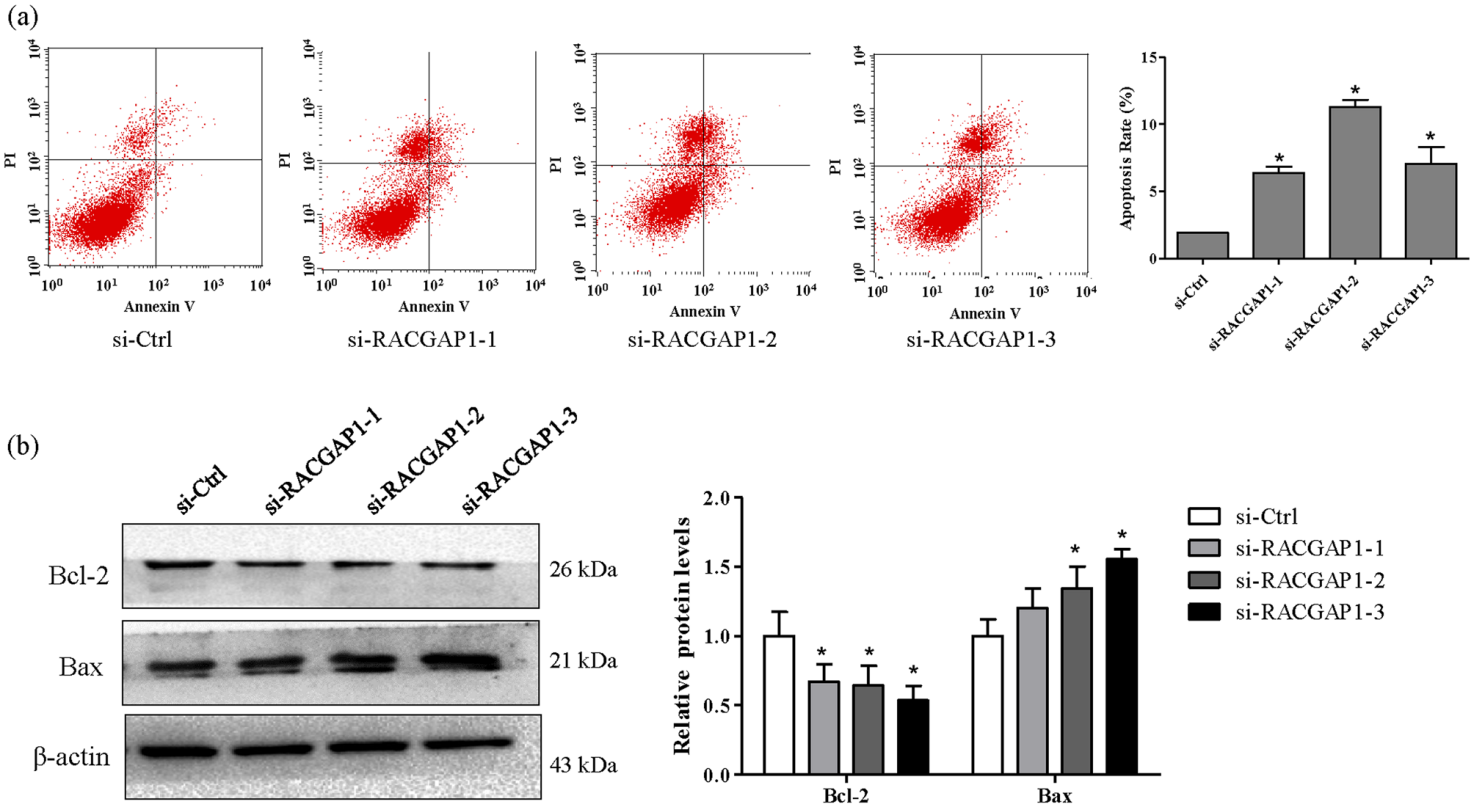
RACGAP1 knockdown induced cell apoptosis in A549 cells. A549 cells were transfected with si-Ctrl or three independent siRNAs for si-RACGAP1. (**a**) Cell apoptosis was determined using Annexin V/PI staining and FACS analysis. Representative plots and percentage of apoptotic cells are presented. *P < 0.05 vs. si-Ctrl. (**b**) Protein levels of Bax and Bcl-2 after RACGAP1 knockdown were determined using Western blot analysis. Representative images are shown and quantitated using ImageLab software. β-actin was used as an internal control. *P < 0.05 vs. si-Ctrl.

To investigate the effects of RACGAP1 on cell apoptosis, Annexin V-647 and PI were used to stain apoptotic cells. As shown in Fig. 8, RACGAP1 knockdown significantly increased the percentage of apoptotic cells (Fig. 8a). Mechanistically, Bcl-2 protein levels were downregulated following RACGAP1 knockdown, whereas Bax expression in si-RACGAP1-2 and si-RACGAP1-3 were significantly upregulated (Fig. 8b).

### RACGAP1 knockdown inhibits PI3K/AKT pathway in A549 cells

**Figure 9:**
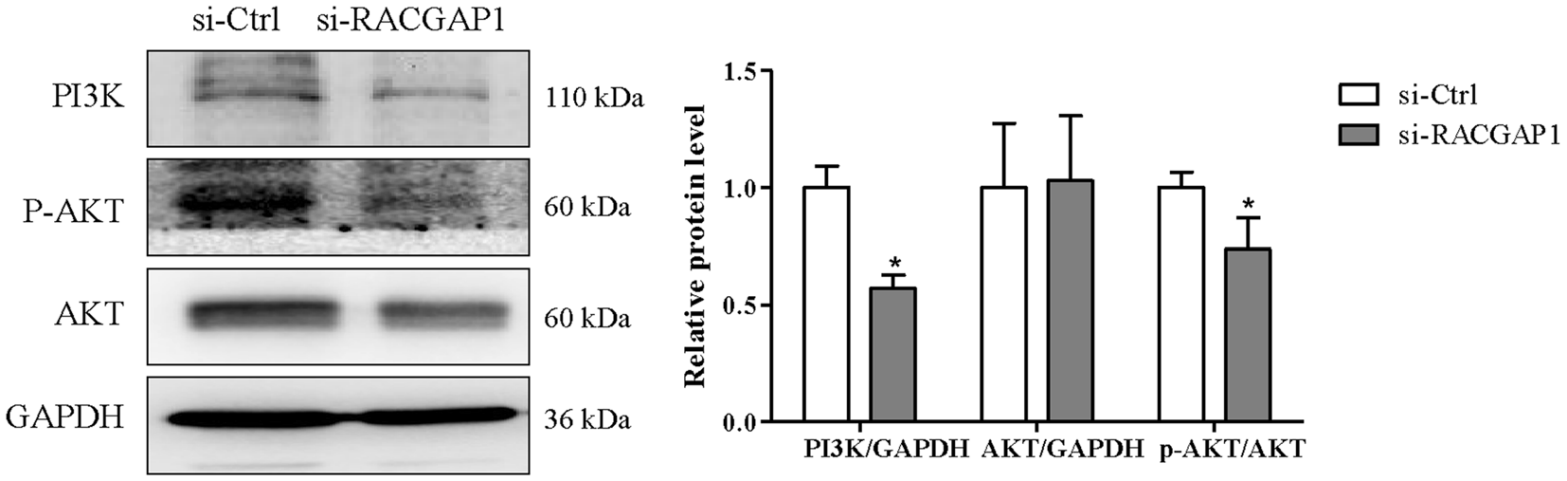
RACGAP1 knockdown inhibited the PI3K/AKT signaling pathway in A549 cells. A549 cells were transfected with si-Ctrl or si-RACGAP1. Protein levels of PI3K, p-AKT, and AKT after RACGAP1 knockdown were determined using Western blot analysis. Representative images are shown and quantitated using ImageLab software. GAPDH was used as an internal control. *P < 0.05 vs. si-Ctrl.

To investigate whether RACGAP1 knockdown can inhibit the PI3K/AKT pathway, we evaluated the expression of PI3K, AKT and p-AKT after RACGAP1 knockdown (si-RACGAP1-2). As shown in Fig. 9, RACGAP1 knockdown significantly decreased PI3K expression and AKT phosphorylation. These findings suggest that RACGAP1 knockdown inhibited proliferation by suppressing PI3K/AKT pathway activation.

## Discussion

Considering its malignant nature, lung cancer has been associated with high morbidity and mortality rates^1,3^. Diagnosis at an early stage, which can dramatically increase cure rates, is important for LUAD treatment. Owing to the poor effects of molecular targeted therapy in advanced-stage lung cancer, the prognosis of LUAD patients remains poor. Therefore, further studies on novel potential biomarker for early diagnosis and prognostic prediction are urgent needed for lung cancer patients. For this purpose, the current study analyzed multiple databases, with our results showing that RACGAP1 mRNA expression was upregulated in multiple tumor tissues (including lung, liver, and colorectal cancers among others) compared to normal adjacent tissues. These findings confirm that RACGAP1 expression was widely upregulated in several types of cancers, including stomach^15^, breast^16,17^ and liver cancers^20^, suggesting that RACGAP1 upregulation might be a common occurrence and plays an essential role in the development and progression of cancers.

Consistent with our finding on mRNA levels, we also confirmed that RACGAP1 protein expression was significantly upregulated in LUAD tissues compared to normal lung tissues. However, more clinic samples need to be analyzed to further verify RACGAP1 expression in future studies. Furthermore, we used the cBioPortal database to analyze genetic variations of RACGAP1 in LUAD, from which we identified four different genetic variations of RACGAP1, including missense mutation, truncating mutation, amplification, and deep deletion. These results indicate that RACGAP1 could potentially play a role of in LUAD diagnosis. Moreover, we analyzed the relationship between RACGAP1 expression and clinical pathological parameters of LUAD patients. Notably, our findings showed that RACGAP1 was significantly correlated with male sex, smoking, stage, nodal metastasis, and TP53 mutant status in LUAD patients, a finding consistent with those presented in previous study on breast and ovarian cancers^24,25^. Our survival analysis based on the Kaplan–Meier Plotter online database and GEPIA database revealed that increased RACGAP1 mRNA expression was associated with poor OS and PFS. The current study found that silencing of RACGAP1 expression in A549 cells using three different siRNAs inhibited cell growth as evidenced by decreased cell viability and colony number. Moreover, our analysis revealed that RACGAP1 knockdown blocked G0/G1 progression downregulated the expression of cyclinD1 and CDK4 in A549 cells. Furthermore, the anti-apoptotic protein Bcl-2 and pro-apoptotic protein Bax, which both belong to the Bcl-2 family, have been found to be critical regulators of this pathway^26^. Notably, we observed that RACGAP1 knockdown increased A549 cell apoptosis. Our analysis of protein expression levels showed that RACGAP1 knockdown downregulated Bcl-2 but upregulated Bax. These findings demonstrated that disruptions in the balance between proliferation and apoptosis in the development of LUAD could be attributed to RACGAP1 overexpression.

The PI3K/AKT signaling pathway is a pivotal cellular signaling cascade that exerts profound regulatory influence over fundamental cellular processes including proliferation, growth, survival, and metabolism^27^. Activated AKT orchestrates cellular proliferation and growth through multiple pathways. It directly influences cell cycle regulatory proteins, facilitating progression through S and G2/M phases. Additionally, AKT modulates protein synthesis and cell size to promote cellular growth. Furthermore, AKT functions as a crucial regulator of cell survival and apoptosis inhibition. Through modulation of signaling molecules such as the Bcl-2 family and caspases, AKT suppresses apoptosis and promotes cell viability, thereby maintaining cellular homeostasis^28^. Therefore, we found that RACGAP1 knockdown decreased the protein expression of PI3K and p-AKT, it had only minor effects on the total expression of AKT. The suggests that suppression of PI3K/AKT pathway might be among the underlying mechanisms behind the suppression of LUAD cell growth by silencing of RACGAP1.

In summary, the current study analyzed RACGAP1 mRNA and protein expression in multiple cancers, including lung cancer, and determined their correlation with prognosis and clinicopathological features. Overall, we found that RACGAP1 knockdown exerted anti-tumor activity in vitro, indicating that RACGAP1 might be involved in lung cancer development. Nonetheless, the underlying regulatory mechanisms still need more explored.

## Materials and methods

### Materials and reagents

The antibody against RACGAP1 (13739-1-AP) was purchased from Proteintech Group (Wuhan, Hubei, China). Antibodies against CDK4, CyclinD1, Bcl-2, Bax, AKT, p-AKT, PI3K, and β-actin were purchased from Cell Signaling Technology (Danvers, MA, United States). Fetal bovine serum, Trypsin–EDTA (0.25%), BCA Protein Assay Kit, and FxCycle PI/RNase Staining Solution were purchased from Thermo Fisher Scientific (Carlsbad, CA, USA). The antibody against GAPDH and the Annexin V 647 Apoptosis Detection Kit and CCK8 kit were obtained from Abbkine (Wuhan, Hubei, China).

### Bioinformatics analysis

GEPIA (http://gepia.cancer-pku.cn/) was performed to analyze the expression levels of RACGAP1 in different types of cancers and overall survival analysis of RACGAP1 proteins from TCGA and Genotype-Tissue Expression, especially lung cancer, including LUAD and LUSC, and paired normal tissues. Changes in RACGAP1 mRNA levels in LUAD/LUSC and healthy tissues were further verified using the UALCAN online database. The protein expression of RACGAP1 in LUAD samples was analyzed through HPA and UALCAN databases.

### Immunohistochemistry-based TMA

The lung cancer samples and matched noncancerous lung tissues (cat no. HLugA150CS03), containing 75 pairs of lung adenocarcinoma tissues and adjacent noncancerous lung tissues, were used for TMA construction. TMAs were created through a contract service at Shanghai OUTDO Biotech, China. The RACGAP1 antibody (1:3000; cat no. 13739-1-AP; Proteintech Group, Wuhan, China) was used to determine protein expression levels. Human tissues were stained using the EliVision Plus Kit (Maixin, China) according to the manufacturer’s instructions. The RACGAP1 immunostaining score was calculated as the sum of the staining intensity score and positive staining cell rate score. The staining intensity was scored as follows: no staining: 0, weak staining: 1, moderate staining: 2, and strong staining: 3. The positive staining cell rate was scored as follows: 0% to 5%: 0, 5% to 25%: 1, 26% to 50%: 2, 51% to 75%: 3, and > 75%: 4. The Wilcoxon test (raw scores) was applied to determine the significance of RACGAP1 staining in primary lung tumors relative to the matched adjacent tumoral tissues^29,30^.

### Genetic alteration analysis

The cBioPortal database was used to obtain information on the genetic variations of RACGAP1 in 2068 cases retrieved from 7 studies (35 cases from MSKCC; 230 cases from TCGA pub; 566 cases from TCGA, PanCancer; 302 cases from OncoSG, 2020; 516 cases from TCGA; 183 cases from Broad; and 0 cases from TSP).

### Clinicopathologic features

The UALCAN database was also used to investigate the relationship between RACGAP1 expression levels and different clinicopathological parameters, which included age, male sex, smoking, stage, nodal metastasis, and TP53 mutant status (summarized in Table 1).

### Survival analysis

The Kaplan‑Meier plotter (www.kmplot.com), containing gene expression data and survival information of patients with LUAD, was queried to analyze the association between RACGAP1 mRNA expression and survival of patients with LUAD, including OS, PFS and PPS. The expression of RACGAP1 among the samples was divided into high or low groups according to the median expression, and the association of RACGAP1 expression with the survival of patients with LUAD was analyzed using the log‑rank test method.

### Cell line and culture

The lung carcinoma cell line A549 was acquired from the Cell Bank of the Chinese Academy of Sciences (Shanghai, China). Cells were maintained in RPMI-1640 medium (Thermo Fisher Scientific) supplemented with 10% fetal bovine serum (Thermo Fisher Scientific), penicillin (100,000 U/L), and streptomycin (100 mg/L) at 37 °C in a 5% CO_2_ atmosphere. The cell lines were tested and authenticated.

### Cell transfection

Three different siRNAs, namely anti-RACGAP1 (si-RACGAP1-1/-2/-3) and control siRNA (si-Ctrl), were purchased from Ribobio (Guangzhou, Guangdong, China). Cells were transfected with siRNAs or si-Ctrl at a concentration of 100 nM using Lipofectamine RNAiMax (Thermo Fisher Scientific) for 6–8 h according to manufacturer’s instructions. The cells were then cultured with complete medium at 37℃ in a humidified atmosphere with 5% CO_2_ for the indicated time points prior to use for experiments.

### Western blot analysis

Cells were harvested and lysed in RIPA lysis buffer containing 1 mM phenylmethylsulfonyl fluoride and protease inhibitors. The BCA Protein Assay Kit was used to measure the concentrations of total protein. Thereafter, 50 µg of total protein lysate was separated on 10% SDS polyacrylamide gel and transferred to polyvinylidene fluoride (PVDF) membranes. Next, the membranes were blocked with 5% skim milk in TBST at room temperature for 2 h, incubated overnight at 4 °C with primary antibodies (all 1:1000), followed by incubation with a horseradish peroxidase-conjugated goat anti-rabbit secondary antibody (1:5000). Proteins were visualized using an enhanced chemiluminescence (ECL) imager (Thermo Fisher Scientific), and band intensities were quantified using ImageLab software. β-actin expression was used as control. Three independent experiments were performed for each assay.

### Cell counting kit-8 (CCK-8) assay

Transfected cells were re-seeded into 96-well plates (2000 cells per well) and cultured at 37 °C and 5% CO_2_ for the indicated time points. CCK-8 reagent (10 µL; Abbkine, Wuhan, Hubei, China) was added to each well. Thereafter, the plates were incubated for an additional 2 h at 37 °C, and the optical density (OD) was measured at a wave length of 450 nm. Cell viability was calculated based on the OD for each group.

### Colony formation assay

Transfected cells were seeded into 12-well plates at a density of 500 cells per well and cultured at 37 °C and 5% CO_2_ for 10–14 days. Cells were then fixed in 4% paraformaldehyde for 20 min and stained with 0.1% crystal violet for 20 min at room temperature. Colonies were counted manually. Each assay was performed in triplicate.

### Cell cycle analysis

Transfected cells were collected and fixed with 70% ethanol at 4 °C overnight. The fixed cells were centrifuged at 2000 rpm for 3 min, washed, and incubated with a mixture of FxCycle PI/ RNase Staining Solution for 30 min at room temperature. Fluorescence-activated cell sorting (FACS; Becton Dickinson, CA, United States) was used to analyze cell cycle progression using ModftLT version 3.0 (Verity Software House Inc., Topsham, ME, USA). The results were the average of three independent experiments.

### Cell apoptosis analysis

Transfected cells were collected and washed twice with ice-cold phosphate-buffered saline and then incubated with Annexin V 647 Apoptosis Detection Kit solution (Abbkine, Wuhan, Hubei, China). The apoptosis rate was analyzed using FACS. The results were the average of three independent experiments.

### Statistics analysis

Data were analyzed using SPSS 22.0 software. GEPIA was performed to determine the association between RACGAP1 expression and survival of LUAD patients. Kaplan–Meier survival curves were plotted for low- and high-expression groups and then analyzed using the log-rank test. Student’s t-test or the Mann–Whitney *U* was used for comparisons between two groups. One-way analysis of variance or the Kruskal–Wallis H test was used for multiple group comparisons. All quantitative data were presented as mean ± standard error of the mean. A *P* value of < 0.05 (two sided) indicated statistical significance. All experiments were performed at least three times.

## Data Availability

The datasets used and/or analysed during the current study are available from the corresponding author on reasonable request.
